# Satisfaction of primary care physicians towards initiation of phone consultations during the COVID-19 pandemic management in Qatar: a cross-sectional study

**DOI:** 10.1186/s12875-022-01654-6

**Published:** 2022-03-12

**Authors:** Marwa Neshnash, Nourhan Metwally, Mansoura Ismail, Anwar Joudeh, Ayman Al-Dahshan, Anna Ramish Sharif, Najma Sharief, Muna Nur, Nagah Selim

**Affiliations:** 1 Primary Health Care Corporation, Doha, Qatar; 2 Family Medicine Department, Faculty of Medicine, Suez Canal University, Ismailia, Egypt; 3 Internal Medicine Department, Al-Khor hospital, Hamad Medical Corporation, Doha, Qatar; 4 Internal Medicine Department, Faculty of Medicine, University of Jordan, Amman, Jordan; 5 Department of Medical Education, Community Medicine Residency Program, Hamad Medical Corporation, Doha, Qatar; 6 Public health department, Faculty of Medicine, Cairo University, Cairo, Egypt

**Keywords:** Telephone consultation, Primary healthcare, COVID-19, Primary care physicians

## Abstract

**Objectives:**

To assess primary care physicians’ satisfaction towards initiation of phone consultation during COVID-19 pandemic management in Qatar and to identify the factors associated with dis/satisfaction.

**Design:**

A cross-sectional web-based survey was conducted from 1 June to 30 July 2020.

**Setting:**

All the available 27 public primary healthcare centers in Qatar at the time of the study.

**Participants:**

Two hundred ninety-four primary care physicians working in the publicly run primary healthcare corporation in Qatar.

**Primary outcome measures:**

Overall satisfaction of primary care physicians with the initiation of phone consultation during the COVID-19 pandemic in Qatar and their satisfaction towards each aspect of this management.

**Results:**

Two hundred thirty-nine primary care physicians participated in the survey with a response rate of 53.1%. Overall, about 45% and 21% of respondents agreed that COVID-19 disease management has highly impacted and very highly impacted their daily practice, respectively. More than half of the physicians (59.9%) indicated being satisfied/highly satisfied with the initiation of telephone consultation service. On the other hand, few physicians were satisfied (14.3%) or highly satisfied (3.4%) with conducting telephone consultations with patients who lack previous electronic medical records. Also, only 20.3% and 3.8% of physicians were satisfied and highly satisfied with the lack of physical examination in telephone consultations, respectively. On bivariate analysis, primary care physicians’ age was significantly associated with the perceived level of impact of COVID-19 management on daily practice (*P* = 0.03). There was no significant association between participants’ characteristics and the level of satisfaction toward telephone consultations. On the other hand, there was a statistically significant association between physicians’ age (*p* = 0.048) and gender (*p* = 0.014) and their level of satisfaction toward communication and support.

**Supplementary Information:**

The online version contains supplementary material available at 10.1186/s12875-022-01654-6.

## Introduction

Coronavirus disease 2019 (COVID- 19) was declared by the WHO as a global pandemic in March 2020 [1]. With the pandemic requiring physical distancing, it was time to leverage the technology available to allow the provision of patient care regardless of their physical location. Hence, telemedicine reformed health care services provided worldwide to enforce physical and social distances [2, 3]. Patient care is an ongoing need and virtual consultations wherever possible have been the magic solution to minimize the potential spread of COVID-19 to protect patients and health professionals from the virus [4]. Cooperation between health service providers, technology expertise, and governments facilitated the provision of a safe, healthy, and accessible way for patients’ care as an alternative to face-to-face consultations keeping a safe environment for the physicians as well and resulting in the emergence of telemedicine [5, 6].

Telephone Consultation, a form of telemedicine has been activated after a long time of negligence and disregard for nearly a half-century ago [7]. Nevertheless, there are several drawbacks to telemedicine. Telephone Consultation is a distinguishing alternative to focus on patients’ history including symptoms of acute illness or follow-up for chronic diseases; however, the patients are not physically assessed [8]. Therefore, health care providers cannot use visual cues or identify abnormal signs in the examination as part of the consultation in patients’ management plan [9]. In addition, telephone consultation is not suitable for acute and emergency cases and language barrier remains a major concern for physicians and patient communications. These factors affect the quality of health information and pose organizational and bureaucratic difficulties [10]. However, telephone consultation prospered during the COVID-19 pandemic due to its ability to deliver the essential healthcare service to patients at a distance, especially for high-risk patients.

The first case of SARS-CoV-2 infection in Qatar was reported on 27 February 2020. In March 2020, the government of Qatar declared a state of emergency with national restrictions after increasing the number of cases [11]. Primary Health Care Corporation (PHCC) started a new avenue of care with the implementation of telemedicine capabilities. Through both telephone and video consultations, patients can now see their physicians from the safety and comfort of their homes. This technology allows for an alternative method to deliver care for patients, which benefits all and especially those at the highest risk of contracting the virus. The move to remote consultation has been successful in implementation in many countries [12, 13] but concerns about collateral effects have been raised due to postponement of “regular care” and loss of contact with vulnerable groups like the elderly, those with chronic diseases, pregnant ladies and children [14].

Telephone consultation service started as an urgent response. However, since the introduction of telephone consultation in a formal way is relatively new, it is important to evaluate this new initiative. In 2014, a qualitative study reported that clinicians’ acceptance was the key to sustainable telehealth services [15]. This study aimed to investigate primary health care physicians (PCPs) satisfactions’ towards initiation of phone consultation during COVID-19 pandemic management in Qatar and to identify the factors associated with dis/satisfaction.

## Methods

### Study setting and population

Qatar is one of the Arabic Gulf countries. The total population is almost 2,947,566 (2021) [16] with the majority living in Doha, the capital of the country, currently served by 29 primary healthcare centers, where 4000 + clinical staff are providing public primary healthcare for the community. Telephone consultation service was started in March 2020 across all PHCC health centers in both general and specialized clinics. Video consultations were started shortly after that in ten health centers and an inbound community call center for general medicine, dental, ophthalmology, dermatology, and physiotherapy services. Patients were triaged through telephone by trained nurses into routine, priority within PHCC scope, and emergency cases and were given appointments or referred to walk-in clinics or emergency departments accordingly. Clinical practice guidelines on telephone consultation were quickly prepared and shared with PCPs who were also trained on the process of telephone consultation. The time allocated to each telephone/video consultation was similar to face-to-face consultations that is 15 min in all clinics. During the early stages of the COVID-19 pandemic (April to July 2020), more than 740,000 telephone consultations were conducted in PHCC-Qatar that constituting around 50% of all consultations [17].

### Study design

This is a cross-sectional web-based survey conducted from June to July 2020. Both the STROBE and CHERRIES guidelines were used for reporting (see supplementary file).

We contacted PHCC operations office for an updated list of the working PCPs during the period of the study. PCPs who were only providing administrative work or were on leave during the data collection period were excluded from the study. They provided a list of 450 PCPs who were mainly family physicians (certified in family medicine) and general practitioners (medical doctors that do not have specialty certification or post-graduate specialized training), as well as a few PCPs who were specialized in internal medicine, pediatrics, ophthalmology, and Ear, Nose and Throat (ENT) physicians. There was no sampling where all of the 450 PCPs were invited to participate in the study through their registered PHCC email.

The survey used in this study was a closed survey as the access to respond was secured by sending it through their formal working email address. This email explained the study aim, a voluntary agreement to participate in the study, expected time to fill the questionnaire, and a link containing anonymous, self-administered questionnaires using the software *SurveyMonkey* without offering incentives. To reduce response bias, a reminder was sent weekly through PHCC operational office through the corporation intranet for five consecutive weeks. Results from all of the respondents were included in the analysis. Apart from the IP address of the respondent, we did not collect any respondent identification information (name or date of birth), and all data collected by the study was secured in password-protected files that only members of the research team had access to it.

### Method of data collection tool

A well-structured questionnaire was developed after an extensive review of the relevant literature on job satisfaction surveys of physicians and was modified according to the local management guidelines at the level of primary healthcare in Qatar [18–20]. It was pretested with a convenient sample of the study population, and some questions were modified based on the feedback. It was validated and reviewed independently by three senior consultant family medicine physicians with research experience. The reliability of the questionnaire was evaluated using Cronbach’s alpha, and the result was a = 0.817. The questionnaire contained the following sections: demographic and professional characteristics including age, gender, years of experience, and qualifications; assessment of PCPs’ satisfaction towards initiation of phone consultation, institutional support as well as the impact of COVID-19 disease on PCPs’ daily practice. We used closed-type questions with multiple-choice options. Response to each question in the questionnaire was devised using Likert scale that provides 5-options for respondent (1 = highly unsatisfied; 2 = unsatisfied; 3 = neutral; 4 = satisfied; 5 = highly satisfied [21]. Likert scale was considered in this study to avoid nonresponse to the questions. Survey questions were not randomized or adaptive to responses. Each screen had only one question with a submit and back buttons to allow for reviewing and modifying the answers before final submission. However, a response was required for each question to proceed to the next screen with a total of 8 screens. Timeframe to submit the survey was not specified but once the survey was submitted, respondents could not log back to the survey website. In order to avoid duplicate responses, unique visitors were identified based on the IP addresses without using cookies or log file analysis. We did not offer any incentive or monetary for participants to undertake the survey.

### Data management plan

The data was entered into a constructed database using the IBM SPSS Statistics for Windows (version 23, IBM Corp., Armonk, N.Y., USA), which was also used for data analysis. Both descriptive and analytic statistics were applied. For the descriptive statistics, summarization using frequency distribution tables and percentages were done for qualitative categorical variables. For analytical statistics, the Fisher’s Exact test (X2) was applied for categorical outcomes. Missing data in this study was less than 1% and no statistical correction was done.

## Results

Out of the 450 physicians invited to participate, 335 visited the first page of the survey (view rate 69.7%), 244 agreed to participate (participation rate 95.7%), and 239 completed the study questionnaire (completion rate 97.9%) giving an overall response rate of 53.1% of the target population. Table 1 shows the background characteristics of the study participants. Almost half (51.9%) of the respondents were aged 41–50 years old; 58.6% were males, and almost two-thirds (66.1%) were family medicine physicians. Most of the participants (77.0%) had more than ten years of experience in general practice.

**Table 1:** Background characteristics of study participants (*N* = 239)

Variable	N (%)
Age (years)	
≤ 40	61 (25.5)
41–50	124 (51.9)
≥ 51	54 (22.6)
Gender	
Female	99 (41.4)
Male	140 (58.6)
Specialty	
Family medicine	158 (66.1)
General practitioner	38 (15.9)
Internal medicine	12 (5.0)
Pediatrics	15 (5.4)
Others specialties	18 (6.6)
Job degree at Primary Health Care Corporation	
General practitioner	44 (18.4)
Specialist	45 (18.8)
Consultant	117 (49.0)
Senior consultant	18 (7.5)
Years of experience	
≤ 5 years	18 (7.5)
6–10 years	37 (15.5)
> 10 years	184 (77.0)

Table 2 displays the level of impact of COVID-19 disease management on PCP’s daily practice. Overall, about 45% and 21% of respondents agreed that COVID-19 disease management has highly impacted and very highly impacted their daily practice, respectively. Many physicians indicated that the COVID-19 pandemic has highly impacted (48%) or very highly impacted (24.3%) their daily practice by causing an unusual strain. Moreover, the absence of physicians due to illness or redistribution between health centers has highly impacted (37.7%) or very highly impacted (13%) physicians’ daily practice. Few respondents indicated that the absence of receptionists due to illness or redistribution between health centers has highly impacted (23.8%) or very highly impacted (11.7%) their daily practice.

**Table 2:** Frequency distribution of the level of impact of COVID-19 disease management on primary care physician’s daily practice (*N* = 239)

Variable	Very high impact	High impact	Some impact	Little impact	No impact at all
	n (%)	n (%)	n (%)	n (%)	n (%)
Unusual strain at your daily practice	58 (24.3)	115 (48.1)	53 (22.2)	7 (2.9)	6 (2.5)
Absence of physicians due to illness or redistribution between health centers	31 (13.0)	90 (37.7)	82 (34.3)	30 (12.6)	6 (2.5)
Absence of nurses due to illness or redistribution between health centers	26 (10.9)	84 (35.1)	87 (36.4)	34 (14.2)	8 (3.3)
Absence of receptionists due to illness or redistribution between health centers	28 (11.7)	57 (23.8)	94 (39.3)	45 (18.8)	15 (6.3)
Limited visits for patient with chronic medical illnesses	31 (13.0)	73 (30.5)	99 (41.4)	29 (12.1)	7 (2.9)
Decrease in patients’ volume	26 (10.9)	73 (30.5)	102 (42.7)	25 (10.5)	13 (5.4)
Patients who can not use Telemedicine	23 (9.6)	82 (34.3)	107 (44.8)	22 (9.2)	5 (2.1)
Rising family and economic concerns among patients	33 (13.8)	103 (43.1)	87 (36.4)	13 (5.4)	3 (1.3)
Rising family and economic concerns among PHCC staff	50 (20.9)	95 (39.7)	78 (32.6)	13 (5.4)	3 (1.3)
Overall impact of COVID-19 disease management on your daily practice	49 (20.5)	107 (44.8)	76 (31.8)	6 (2.5)	1 (0.4)

Table 3 shows the level of physicians’ satisfaction toward the application of telephone consultations during the COVID-19 pandemic. Overall, most respondents (59.9%) indicated being satisfied/highly satisfied with the telephone consultation service. For instance, 68.3% of respondents were satisfied/ highly satisfied with the time allocated for a telephone consultation. Similarly, 64.6% reported being satisfied/highly satisfied with patient identification in telephone consultations. On the other hand, few physicians were satisfied (14.3%) or highly satisfied (3.4%) with conducting telephone consultations with patients who lack previous electronic medical records. Also, only 20.3% and 3.8% of physicians were satisfied and highly satisfied with the lack of physical examination in telephone consultations, respectively.

**Table 3:** Primary care physicians’ satisfaction toward the application of telephone consultations during COVID-19 pandemic (*N* = 239)

Variable	Highly satisfied	satisfied	Neutral	Unsatisfied	Highly Unsatisfied
	n (%)	n (%)	n (%)	n (%)	n (%)
Process of telephone consultation booking	38 (16.0)	101 (42.6)	75 (31.6)	16 (6.8)	7 (3.0)
Time allocated for telephone consultation	34 (14.3)	128 (54.0)	61 (25.7)	11 (4.6)	3 (1.3)
Patient identification in telephone consultations	31 (13.1)	122 (51.5)	63 (26.6)	15 (6.3)	6 (2.5)
Ensuring patient confidentiality with telephone consultations	29 (12.2)	108 (45.6)	78 (32.9)	16 (6.8)	6 (2.5)
Absence of physical contact with patients to reduce the risk of infection	67 (28.3)	104 (43.9)	53 (22.4)	11 (4.6)	2 (0.8)
Lack of physical examination in telephone consultations	9 (3.8)	48 (20.3)	115 (48.5)	59 (24.9)	6 (2.5)
Having telephone consultations with patients with no previous electronic medical records	8 (3.4)	34 (14.3)	80 (33.8)	89 (37.6)	26 (11.0)
Your training on performing telephone consultation	30 (12.7)	101 (42.6)	72 (30.4)	20 (8.4)	14 (5.9)
Overall satisfaction on telephone consultation service	35 (14.8)	107 (45.1)	86 (36.3)	7 (3.0)	2 (0.8)

Table 4 displays the level of physicians’ satisfaction toward communication and support during the COVID-19 pandemic. Almost a quarter of physicians (75.7%) were satisfied/ highly satisfied with the availability of their supervisor to provide help when needed. Similarly, 71.6% indicated being satisfied/highly satisfied with the ability of supervisors to address their questions and concerns. On the other hand, few physicians were satisfied (14.3%) or highly satisfied (3.4%) with PHCC’s staff support hotline.

**Table 4:** Primary care physicians’ satisfaction toward communication and support during Covid-19 pandemic (*N* = 239)

Variable	Highly satisfied	satisfied	Neutral	Unsatisfied	Highly Unsatisfied
	n (%)	n (%)	n (%)	n (%)	n (%)
Communication between higher management and employee	29 (12.1)	104 (43.5)	71 (29.7)	19 (7.9)	16 (6.7)
Communication between health center management and staff	44 (18.4)	118 (49.4)	53 (22.2)	11 (4.6)	13 (5.4)
Supervisor’s effort to promote a positive teamwork atmosphere	45 (18.8)	124 (51.9)	50 (20.9)	10 (4.2)	10 (4.2)
The ability of your supervisor to address your questions and concerns	47 (19.7)	124 (51.9)	48 (20.1)	11 (4.6)	9 (3.8)
Availability of your supervisor to provide help when needed	56 (23.4)	125 (52.3)	44 (18.4)	6 (2.5)	8 (3.3)
Availability of resources to support your decision making about patient care	36 (15.1)	122 (51.0)	61 (25.5)	10 (4.2)	10 (4.2)
Primary Health Care Corporation’s staff support hotline	24 (10.0)	86 (36.0)	96 (40.2)	21 (8.8)	12 (5.0)
Overall satisfaction on communication and support	40 (16.7)	101 (42.3)	72 (30.1)	13 (5.4)	13 (5.4)

Table 5 describes the association between PCPs’ perceived impact of COVID-19 management on daily practice their background characteristics. On bivariate analysis, PCPs’ age was significantly associated with the perceived level of impact of COVID-19 management on daily practice (*P* = 0.03). However, gender and years of experience were not associated with the outcome measures.

**Table 5:** Perceived impact of COVID-19 management on daily practice by age, gender, and duration of clinical practice (*N* = 239) (Fisher’s Exact Test)

Variable	High/very high impact	Neutral	Little/ no impact	*p*-value
	n (%)	n (%)	n (%)	
Age (years)				0.030*
≤ 40	49 (80.3)	12 (19.7)	0 (0.0)	
41–50	77 (62.1)	42 (33.9)	5 (4.0)	
≥ 51	30 (55.6)	22 (40.7)	2 (3.7)	
Gender				0.817
Female	67 (67.7)	29 (29.3)	3 (3.0)	
Male	89 (63.6)	47 (33.6)	4 (2.9)	
Years of experience				
≤ 10 years	38 (69.1)	16 (29.1)	1 (1.8)	
> 10 years	118 (64.1)	60 (32.6)	6 (3.3)	0.793

Table 6 shows a comparison between participants’ characteristics (age, gender, and duration of clinical practice) and the level of satisfaction toward telephone consultations (above) and communication and support (down). As shown in Table 6, there was no significant association between participants’ characteristics and the level of satisfaction toward telephone consultations. On the other hand, there was a statistically significant association between physicians’ age (*p* = 0.048) and gender (*p* = 0.014) and their level of satisfaction toward communication and support.

**Table 6:** The level of satisfaction toward telephone consultations by age, gender, and duration of clinical practice (*N* = 239) (Fisher’s Exact Test)

Variable	Satisfied / highly satisfied	Neutral	Unsatisfied / highly unsatisfied	*p*-value
	n (%)	n (%)	n (%)	
Satisfaction toward telephone consultations				
Age (years)				0.960
≤ 40	35 (58.3)	22 (36.7)	3 (5.0)	
41–50	74 (60.2)	44 (35.8)	5 (4.0)	
≥ 51	33 (61.1)	20 (37.0)	1 (1.9)	
Gender				0.863
Female	57 (58.2)	37 (37.8)	4 (4.0)	
Male	85 (61.2)	49 (35.3)	5 (3.6)	
Years of experience				
≤ 10 years	31 (56.4)	23 (41.8)	1 (1.8)	
> 10 years	111 (61.0)	63 (34.6)	8 (4.4)	0.518
Satisfaction toward communication and support				
Age (years)				0.048*
≤ 40	32 (52.5)	22 (36.1)	7 (11.5)	
41–50	68 (54.8)	39 (31.5)	17 (13.7)	
≥ 51	41 (75.9)	11 (20.4)	2 (3.7)	
Gender				0.014*
Female	51 (51.5)	40 (40.4)	8 (8.1)	
Male	90 (64.3)	32 (22.9)	18 (12.9)	
Years of experience				
≤ 10 years	32 (58.2)	17 (30.9)	6 (10.9)	
> 10 years	109 (59.2)	55 (29.9)	20 (10.9)	0.974

## Discussion

Primary care is an essential foundation for the global response to coronavirus disease-2019 (COVID-19). Evidence since the start of the pandemic has shown that the role of primary care during a public health emergency leans primarily to maintaining essential health services and secondly to responding to the outbreak [22].

The State of Qatar has taken multiple precautionary measures to prevent the spread of COVID-19 in order to ensure the safety of all its residents. Telephone consultation service is one of these important measures [23].

Telephone consultation by family physicians has been around for some time. It has particularly been on the rise as the demand for GP appointments increased. For instance, statistics showed that in 1995, telephone consultations made up 3% of all consultations in the UK and this rose to 12% in 2009 [24]. A more recent survey carried out by the Royal College of General Practitioners during the pandemic (July 2020) showed that 61% of GP appointments were conducted by telephone [25].

The current study assessed the impact of COVID-19 pandemic management on the daily practice in health centers, in addition to physicians’ satisfaction in PHCC toward using telemedicine during the COVID-19 pandemic.

### Impact of COVID 19 on daily practice

Physicians have faced challenges at different levels during the COVID-19 pandemic that affected their daily practice including but not limited to causing an unusual strain on their daily practice, absence of physicians, nurses, or receptionists due to illness or redistribution between health centers, limitation of visits of patients with chronic medical illnesses, inability to use telemedicine by some patients, rising family and economic concerns among patients as well as among PHCC staff. The study showed that many physicians indicated that the COVID-19 pandemic has highly impacted their daily practice and caused an unusual strain, and they also reported that the absence of physicians due to illness or redistribution between health centers has also highly impacted their daily practice.

In a study conducted in Bangladesh, participants indicated that the health sector faced a shortage of medical workers. Moreover, many registered doctors did not practice medicine resulting in a higher workload by the active medical workforce in public as well as in private facilities which might lead to increased mental stress. Medical facilities also had few nurses, who had to work 16–17 h shifts per day. Additionally, fear of infection prevented workers from joining their workplace. The participants were more concerned about family members being infected by them rather than themselves being infected, leading to further mental stress [26].

The impact of COVID19 on daily practice has been felt by general practitioners (GPs) on a global level. In Italy for instance, a study found that an increased number of GPs reported physical and psychological pressures during this pandemic [27]. In comparison to many other countries, the use of telemedicine was limited in Italy, one of the reasons being due to the little connection that existed between different levels of the available telemedicine services. There was also a lack of access to patient records within the national health service there, as well as heavy and impractical privacy regulations hindering its wide use [28]. The higher level of primary care physicians satisfaction with telephone consultations in PHCC in Qatar in the current study could be attributed to the previously established electronic medical records systems for both nationals and expatriates that are also connected to the public secondary and tertiary care sectors in Qatar which facilitated consistency and continuity of care between providers.

Beyond health issues, the impact of the pandemic on the economic and social dynamics of both patients and healthcare workers had further strained the primary healthcare services. In the current study, around 6 in 10 respondents indicated that rising family and economic concerns among patients and PHCC staff had highly impacted or very highly impacted their daily work. Similarly, a survey by Song et al. on 398 practices in Massachusetts found that the COVID-19 pandemic has substantially disturbed the healthcare system where more than 60% of practices stated they would cut salaries of their providers/employees, reduce their services or lay off their employees. Also, 47% of primary care practices forecasted that they will have to close their practice with a reported likelihood averaging 15% [29].

### Physician satisfaction toward communication and team support

Supportive work culture is vital to maintaining the resilience of clinicians during a crisis such as the COVID-19 pandemic. The study showed that most PCPs were satisfied with the availability of their supervisor to provide help when needed and the ability of supervisors to address their questions and concerns. On the other hand, few physicians were satisfied with the available PHCC ‘s staff support hotline. While the study that was done in the united states of America reported that “Dramatic changes in practice have come very quickly, without much prior preparation. Little assistance, or even clear guidance, has come from authorities, and the resulting changes vary widely by practice organizational structure” [30]. Also, the study conducted in Bangladesh showed that the healthcare workers were dissatisfied with some discriminatory initiatives taken up by the authorities. Besides, they did not have any training regarding how to function correctly in a virus outbreak. Consequently, both doctors and patients were unsure about the protocols needed to maintain safety, which further increased the risk of infection [26].

A systemic review of a large number of studies conducted between 2005 and 2015 concluded that telemedicine has a significant role in addressing some of the challenges faced by primary care today [31]. Teamwork within the practice and support from team leaders is vital in achieving this to make telephone consultations an integral part of a primary care setting.

According to Gray and Sanders, facilitating resilience and improving communication between primary care professionals during these challenging times could not be overstated. Professional resilience does not only depend on personal traits and social factors, it is also affected by modifiable workplace characteristics such as support from management, teamwork dynamics, and supportive colleagues [32].

### Physicians' satisfaction toward telephone consultation service

It is without a doubt that the COVID-19 pandemic has reshaped how healthcare services are provided to patients worldwide. As a response to the pandemic and complying with the social and physical distancing, telemedicine has emerged as an essential technology to bring medical care to patients [3].

Nonetheless, telemedicine cannot replace personal medical care in all cases. Telemedicine should not be used in severe or unstable conditions or whenever GPs’ examination is needed. The study showed an overall high satisfaction rate with the teleconsultation service, but only a few physicians were satisfied with the lack of physical examination in a telephone consultation. In addition, the study demonstrated that satisfaction with phone consultations was more among male physicians (41–50 years old). A study conducted in the UAE showed that a previous experience with telemedicine was associated with twofold confidence in treating acute conditions, less than a half of the perceived risk of misdiagnosis, and an increased ability to provide patients with health education and enhance the physician–patient rapport [33]. While the research conducted in Oman showed that many doctors were dissatisfied with communicating by telephone, due to the absence of visual cues, lack of comprehensive assessment, and inability to perform physical examination or share laboratory results [34]. However, Chinese health authorities actively discouraged patients from engaging in face-to-face visits to hospital/outpatient departments unless it was essential but instead encouraged telephone and/or online consultations [35].

In Romania, a study on the opinions of family doctors regarding the use of telemedicine found that more than a quarter of GPs found it easier to address patients’ healthcare needs remotely, while half responded that teleconsultations were time-consuming compared to face-to-face visits [36].

On the other hand, a study conducted in Brazil showed that physicians based in towns with lower GDP per capita and with a higher number of reported cases were less likely to use the service [37].

In California, physicians indicated that telemedicine improved patient access to care by providing greater convenience, although some expressed concern that certain groups of vulnerable patients were unable to navigate or did not possess the technology required to participate in telemedicine visits. Physicians noted that telemedicine visits offered more time for patient counseling, opportunities for better medication reconciliations, and the ability to see and evaluate patient home environments and connect with patient families [38].

Studies before the pandemic had also shown that telephone consultations improved access to groups of patients who would otherwise miss important annual reviews such as asthma reviews. Once such a study was conducted in 2003, whereby a group of asthmatic patients was randomized into two groups, one group had a telephone consultation review and another booked in for face-to-face review. It was evident that the telephone consultations enabled more patients to be reviewed as it was easier, quicker and more convenient for the patients [39].

PHCC in Qatar provides access to patients’ records through a well-designed system that has been available for years. Moreover, this electronic record directly links and allow primary care physician access to hospital records. This greatly aided the smooth transition to telephone consultation in the early days of the pandemic. The majority of the respondents were not only satisfied with the telephone consultation overall, but they were also satisfied with the time allocated for the telephone appointments and the ease of patient identifications through the electronic records over the telephone.

Despite rapid transit to telephone consultation in primary care in Qatar, around half of the respondents indicated that they were satisfied or highly satisfied with their training on conducting telephone consultations. An earlier Cochrane review on training interventions to improve telephone consultations of clinicians found that telephone communications with patients were mostly of low quality, and that undergraduate and postgraduate curricula lacked specific training on telephone consultations [40]. However, a recent mixed-methods study on GP trainees’ experiences on telephone consultations found a strong positive correlation between having prior training on telephone consultation and GP trainees’ confidence (*r*^*2*^ = 0.71, *P* < 0.0001) [41].

### Physicians characteristics and the level of satisfaction toward service changes

The current study showed that the impact of COVID-19 pandemic-related changes in the primary care service in Qatar was higher on younger PCPs. Moreover, younger female PCPs were less satisfied with communication and support within their health centers. However, age, gender, and duration of clinical practice were not associated with PCPs satisfaction with telephone consultations.

A similar study on the psychological impact of the first wave of COVID-19 pandemic on 215 GPs from Italy found that being female was associated with higher exhaustion and depression rates in comparison to male PCPs. In addition, PCPs with longer clinical experiences had lower anxiety and higher professional efficacy rates [42]. Another interesting study by Cai et al. on 534 frontline medical staff in Hubei, China during the initial stages of the pandemic found that younger medical staff were more anxious about infecting their families, whereas older staff were more worried about their own safety and for being exhausted for working long hours. On the other hand, coping strategies such as positive attitude from other colleagues and effective guidance on disease transmission prevention had a larger impact on reducing stress in female staff than in male staff [43].

### Lessons learned from the study

The ongoing COVID-19 pandemic has highlighted the vital role of primary healthcare as an integral component of any successful health system emergency plan. Therefore, it is important to experience deep learning and implement changes to the health system to achieve effective and safe healthcare delivery. Our study findings were largely similar to other studies conducted in different countries and we believe that several lessons could be learned from this experience. First, in addition to the important role of telemedicine in providing healthcare access to the wider community throughout COVID-19 pandemic, it can also provide effective ways to mitigate potential obstacles in primary healthcare during the aftermath of this pandemic. In order to achieve safe and effective telemedicine, healthcare systems should deliver teaching and training programs to meet the professional needs of PCPs, and implement national digital health framework to ensure continuity of care.

The second lesson is that healthcare policymakers should identify the needs of younger and female PCPs in order to reduce the impact of this pandemic on them and set measures to prevent their burnout. Younger and female PCPs should be invited more often frequently to participate in the guidelines’ committees and ensure their accessibility to supportive and coping strategies. The third lesson is that managers and supervisors should promote teamwork and maintain positive working environment. Collaborative work in teams improves the efficacy of responses to any emerging need and reduces the stress experienced by team members.

### Study limitations

This study has several limitations. First, the response rate was lower than desired (53%) due to the difficulties of data collection during the COVID-19 pandemic. Also, there is a possibility for response bias where a self-reported response might not represent actual or genuine answers. Furthermore, as a cross-sectional study, results may not reflect primary care opinions on later stages of the pandemic. A follow-up mixed-method study might allow for an in-depth understanding of primary care physicians' perspective on telemedicine and changes exerted by the pandemic on their daily practice. Despite these limitations, this study represents some of the early evidence on primary care physicians’ perception toward telemedicine in Qatar which allows for data-based planning of service reorganization to manage the pandemic safely and effectively.

## Conclusion

The move to the use of more telephone consultations in PHCC during this pandemic has been both timely and efficient. This was mainly due to the swift decision-making and actions taken in the early days of the pandemic. With a well-built infrastructure from staff to equipment pre-pandemic, it was easy to make the transition. However, research is still needed to assess both PCPs and patients’ satisfaction with telephone consultations, as well as its efficacy and impact on patients, PCPs and the healthcare system.

## Supplementary Information

**Additional file 1. **Checklist for Reporting Results of Internet E-Surveys (CHERRIES).

**Additional file 2. **STROBE Statement—Checklist of items that should be included in reports of  cross-sectional studies.

## Data Availability

All data relevant to the study are included in the article or uploaded as supplementary information.
